# Characterization of variable EST SSR markers for Norway spruce (*Picea abies *L.)

**DOI:** 10.1186/1756-0500-4-401

**Published:** 2011-10-12

**Authors:** Silvia Fluch, Agnes Burg, Dieter Kopecky, Andreas Homolka, Nadine Spiess, Giovanni G Vendramin

**Affiliations:** 1 AIT Austrian Institute of Technology GmbH, Health & Environment Department, Bioresources, 3430 Tulln, Austria; 2 Istituto di Genetica Vegetale, CNR, via Madonna del Piano 10, 50019 Sesto Fiorentino (Firenze), Italy

## Abstract

**Background:**

Norway spruce is widely distributed across Europe and the predominant tree of the Alpine region. Fast growth and the fact that timber can be harvested cost-effectively in relatively young populations define its status as one of the economically most important tree species of Northern Europe. In this study, EST derived simple sequence repeat (SSR) markers were developed for the assessment of putative functional diversity in Austrian Norway spruce stands.

**Results:**

SSR sequences were identified by analyzing 14,022 publicly available EST sequences. Tri-nucleotide repeat motifs were most abundant in the data set followed by penta- and hexa-nucleotide repeats. Specific primer pairs were designed for sixty loci. Among these, 27 displayed polymorphism in a testing population of 16 *P. abies *individuals sampled across Austria and in an additional screening population of 96 *P. abies *individuals from two geographically distinct Austrian populations. Allele numbers per locus ranged from two to 17 with observed heterozygosity ranging from 0.075 to 0.99.

**Conclusions:**

We have characterized variable EST SSR markers for Norway spruce detected in expressed genes. Due to their moderate to high degree of variability in the two tested screening populations, these newly developed SSR markers are well suited for the analysis of stress related functional variation present in Norway spruce populations.

## Background

Natural populations of *Picea abies *L. (Norway spruce) are found from north-western Europe outside permafrost areas down to northern Greece, westwards to the Massif Central (France) and east to the Ural Mountains. *Picea abies *is growing above 400-500 m and ascends close to 2000 m in the Alps. Studies on genetic variation based on allozymes have shown that *Picea abies *genetic differentiation among populations is rather low over its whole distribution range [1,2]. Previous studies on the genetic structure of *P. abies *using organelle markers showed pronounced differentiation between north-east boreal origins and areas in the central European mountains [3,4], supporting the hypothesis of two distinct main glacial refugia as postulated from pollen data [5].

Initial reports on the occurrence of SSRs in conifers such as *Pinus radiata *[6], *Pinus sylvestris *[7] or *Picea abies *[8] have shown that marker development for such complex genomes is difficult. Frequently several DNA fragments in addition to the expected ones are amplified when using primers flanking putative SSR regions. This can be attributed to the very large size of the conifer genomes, with Norway spruce having 39.140 Mb in the diploid genome (2n = 24) as well as the high proportion of repetitive DNA in the conifer genomes [9]. These repetitive elements as well as pseudogenes frequently produce complex amplification products from multiple loci [10,11]. To overcome this problem, Scotti et al. [12] showed that by isolating di-nucleotide SSRs from cDNA libraries of conifer species this problem can be overcome relying on the fact that expressed genes are less likely to be of repetitive nature in the genome.

Following this line of exploiting the advantages of publicly available data on expressed sequences (ESTs) as source for marker development [13] as described also by Rungis et al. [14] for conifers, we used Norway spruce EST sequences from NCBI for in silico identification of SSR regions which could serve for marker development.

## Results

Screening of 14,022 Norway Spruce EST sequences from the NCBI dbEST revealed 158 sequences containing various repeat motifs. Clustering of these sequences produced 92 SSR containing 'unigenes' which fell into 36 clusters and 56 singletons. In these 92 unigenes, 48 different repeat motifs were identified, with tri-nucleotide (50%), in particular (CGG)_n _and CAA)_n_, repeats being the most abundant, followed by penta- (23%) and hexanucleotide repeats (10.4%) respectively. Di- and tetranucleotide repeats were the least abundant with 8.3% each. Twenty-seven out of 60 planned primer pairs amplified polymorphic products in the testing population of 16 individuals, while two did not generate a fragment and 31 proved to be monomorphic [see Additional file 1]. Four of the 27 polymorphic SSR regions showing more than 2 alleles per locus (Pa_16, Pa_24, Pa_34 and Pa_40) in the testing population were excluded from further analysis. In a total of 96 individuals 135 alleles were detected in the 23 remaining loci (Table 1) ranging from 2 to 17 alleles per locus (Table 2) with a mean value of 5.6. Total number of effective alleles was and 50.645 with a minimum of 1.123, a maximum of 4.115 and a mean value of 2.202. 87% of the variable regions were based on trinucleotide repeats, the rest were pentanucleotide repeats. Di-, tetra and hexanucleotide repeats proofed to be not polymorphic. Loci Pa_41 and Pa_53 were not polymorphic in the Mayrhofen population. Values for expected heterozygosity ranged from 0.021 to 0.780 in the Gusswerk population (mean 0.367) and from 0.123 to 0.769 in the Mayrhofen population (mean 0.385). Observed heterozygosity was found to be between 0.021 and 1.000 in the Gusswerk population (mean 0.461) and between 0.042 and 1.000 in the Mayrhofen population (mean 0.515). Differences in observed heterozygosities between the two populations ranged from zero (Pa_44) to 0.894 (Pa_49). The minimum difference of expected heterozygosities between the two populations was found at locus Pa_12 (0.002) and the maximum at locus Pa_49 (0.382). Regarding the Gusswerk population, in eleven loci the observed heterozygosity was significantly higher and in three loci significantly lower than the expected value. In the Mayrhofen population we found thirteen loci with the observed heterozygosity significantly higher than the expected and in four loci it was significantly lower. Eleven loci showed significant departure from Hardy-Weinberg expectations (HWE, Guo and Thompson's exact test [P < 0.05]) within the 48 investigated individuals from the Gusswerk population. The number of loci with significant departure from HWE was higher in the Mayerhofen population (16 loci). Frequency of null alleles was variable from zero to 99.0% in the Gusswerk population and from zero to 85.4% in the Mayerhofen population. In both populations, null alleles were present with a high frequency (above 5%) in more than half of the loci deviating from HWE. Tests for linkage disequilibrium (P < 0.01) revealed no disequilibrium among pair wise compared loci. F_st _ranged between 0 and 0.288 with a mean value of 0.033 (after application of the ENA correction described in Chapuis and Estoup (2007), Table 3). Repeat numbers of all polymorphic di- and pentanucleotide repeats added up to a multiple of three and therefore did not cause a frameshift. All polymorphic SSRs are lying within open reading frame sequences (ORF) as detected by GetORF [15]. Fourteen sequences hit functionally annotated genes when compared to the NCBI nucleotide database [see Additional file 2]. The test for outlier loci revealed that Pa_51 (P = 1.000) and Pa_42 (P = 0.981) are under positive selection while the other loci are supposed to behave neutral (P < 0.95).

**Table 1:** Characteristics of polymorphic SSR loci isolated from *Picea abies *L

Locus name	GeneBank Accession	Repeat type	Primer Sequence (5' - 3')	Size range (bp)	T_a _(°C)
Pa_5	GT887224	(CCG)_n_	F: AGGTGGATATGTTCATCAAC	136-158	53
			R: CGCATACTCATCCCTAAC		
Pa_12	AM170164	(AAG)_n_	F: GACCGAGAACCCTTGTT	136-138	53
			R: CAAAAGCAGGAAAGAAGAAC		
Pa_22	GT886979	(TTC)_n_	F: TCACTGGCCACAGTTTATCG	169-188	55
			R: ATGAGGCCCAAGAGGAAGAC		
Pa_25	GT887533	(CTG)_n_	F: TGATTGAAATGATGGCTGCT	131-165	55
			R: CATGTACGGTGCTCCTCCTC		
Pa_28	GT887962	(TCG)_n_	F: GGCCGAAAGTGCTACTGCTA	148-162	62
			R: TGCTCCAGAAGAACACTCACA		
Pa_29	GT884785	(CAA)_n_	F: ACAACAGCAACAGCAGCAAC	97-108	62
			R: CGGGCTGAAGAATTTGTTGT		
Pa_33	GT884592	(CGG)_n_	F: GGTCGAGGAGGAGGAGGTAG	91-104	62
			R: CACCGCTAGTGCAGTCTCTG		
Pa_36	GT887107	(CGG)_n_	F: CGGCAGGAACATCACTGTTA	178-200	62
			R: ACCGTAACCTCCCCTACCAC		
Pa_41	AM169657	(CTG)_n_	F: CGAAGAAGAAAGCGAGGATG	176-183	62
			R: CAGGCTGCGAGAATCCTCTA		
Pa_42	AM169827	(CCG)_n_	F: CAATGCAATGGCCTCCTATC	160-163	62
			R: TAGACAGCAGCACGTCTCGT		
Pa_43	AM169948	(GAA)_n_	F: AACCAGCCGGAGTCTGTAAA	168-175	62
			R: TGCTTCTGTCTGACCAGGTG		
Pa_44	AM171132	(GGA)_n_	F: AAGGCAGCCAAAGTGAAGAA	274-293	62
			R: CTTGGCATTCCCTAGTGAGC		
Pa_47	AM172144	(CAG)_n_	F: ATCAATTGCCCTACCAGCAC	106-118	62
			R: TGCTCAATTTCCTGCATCTG		
Pa_48	AM172164	(CGG)_n_	F: ATTGCACAAGAGCGAACCTT	131-195	62
			R: CCAGCACCAAAATCACCAG		
Pa_49	AM173566	(ATG)_n_	F: GAAAGGCGAAAGCAAAACTG	117,128	62
			R: CCGTGTTTTTGAGGGAAGAA		
Pa_50	AM174647	(CAG)_n_	F: CCTCTCAGCCAAATGCAAAT	124-129	62
			R: GTCTCCGCAGAGCCATTACT		
Pa_51	AM175215	(CCA)_n_	F: CAGATGTGGGCACTTGTTTG	119-140	62
			R: TGGTCATGGTGGTGTTCAT		
Pa_52	AM175251	(CCA)_n_	F: GTAGTGTCACCGGCTCCTTT	135-145	62
			R: CTGGAGGATGATGGTGGTG		
Pa_53	ES290836	(TCG)_n_	F: CCCAATGAAGATCTCGATGC	176-178	62
			R: GGCTTTGCAGGAAACAAGAA		
Pa_55	GT887161	(GAA)_n_	F: TGAGCAACCAGAAAATCACG	148-158	62
			R: TCTCAACCCCTTTGTTTTCG		
Pa_56	GT884695	(AGGTG)_n_	F: ATCGTCTGCATTGCATTCAC	113-128	62
			R: CTTCGTTCCTTCCTGATCCA		
Pa_59	AM169794	(CTCTG)_n_	F: TAGACCACGTTGTGCTGAAA	90-112	62
			R: GCGACCTTTCGCAATTAAAC		
Pa_60	GT885001	(GGCTG)_n_	F: CGCCGTATCCATTCCCAAGC	241-264	62
			R: CCCAGCCCAGTTCAGTTTGC		

T_a_, annealing temperature

**Table 2:** Number of observed and effective alleles, observed and expected heterozygosity estimates for 23 polymorphic SSR markers with two *Picea abies *L. populations

Locus name	N_a_	N_e_	Gusswerk		Mayrhofen	
			H_o_	H_e_	H_o_	H_e_
Pa_5	7	1.805	0.432	0.347	0.791	0.533
Pa_12	2	1.999	1.000	0.507	0.956	0.505
Pa_22	6	2.016	0.511	0.439	0.892	0.556
Pa_25	8	1.318	0.109	0.185	0.042	0.295
Pa_28	8	4.049	0.681	0.780	0.542	0.428
Pa_29	5	1.792	0.447	0.351	0.688	0.522
Pa_33	9	2.572	0.957	0.637	0.979	0.584
Pa_36	5	1.175	0.149	0.141	0.167	0.159
Pa_41	5	1.286	0.261	0.412	n.p.	n.p.
Pa_42	4	2.311	0.021	0.021	0.255	0.256
Pa_43	6	2.559	0.174	0.162	0.234	0.280
Pa_44	7	1.357	0.188	0.232	0.188	0.299
Pa_47	4	2.052	0.500	0.407	0.979	0.535
Pa_48	17	4.115	0.766	0.779	0.660	0.748
Pa_49	5	2.917	0.979	0.505	0.085	0.123
Pa_50	4	1.959	0.289	0.293	1.000	0.505
Pa_51	3	2.012	0.042	0.041	0.167	0.154
Pa_52	5	2.914	0.917	0.546	0.688	0.470
Pa_53	2	2.000	0.021	0.021	n.p.	n.p.
Pa_55	3	2.009	0.958	0.514	0.896	0.500
Pa_56	6	3.508	0.625	0.655	0.854	0.769
Pa_59	3	1.123	0.042	0.041	0.146	0.175
Pa_60	5	1.799	0.542	0.423	0.646	0.469
Mean	5.6	2.202	0.461	0.367	0.515	0.385
Total	129	50.645				

N_a _number of observed alleles, N_e_, effective number of alleles, H_o _observed heterozygosity, H_e _expected heterozygosity, n.p. not polymorphic

**Table 3:** Estimation of uncorrected and corrected FST, frequency of null alleles and deviation from Hardy-Weinberg equilibrium

Locus name	F_st_	F_st _(ENA)	Gusswerk		Mayrhofen	
			F_n_	HWE p-value	F_n_	HWE p-value
Pa_5	0.046	0.046	0.000	0.340	0.000	0.001*
Pa_12	0.000	0.000	0.000	0.000*	0.000	0.000*
Pa_22	0.065	0.065	0.167	0.038*	0.006	0.000*
Pa_25	0.007	0.031	0.127	0.004*	0.244	0.000*
Pa_28	0.004	0.003	0.191	0.000*	0.274	0.003*
Pa_29	0.035	0.036	0.000	0.088	0.008	0.001*
Pa_33	0.020	0.020	0.000	0.000*	0.000	0.000*
Pa_36	0.000	0.000	0.000	1.000	0.000	1.000
Pa_41	0.007	0.041	0.293	0.000*	0.146	1.000
Pa_42	0.107	0.144	0.990	n.a.	0.854	0.023*
Pa_43	0.000	0.000	0.000	1.000	0.100	0.013*
Pa_44	0.000	0.000	0.060	0.009*	0.120	0.000*
Pa_47	0.000	0.000	0.000	0.212	0.000	0.097
Pa_48	0.000	0.000	0.046	0.074	0.047	0.256
Pa_49	0.000	0.000	0.000	0.000*	0.000	0.000*
Pa_50	0.011	0.023	0.087	0.116	0.000	1.000
Pa_51	0.288	0.288	0.979	1.000	0.571	0.000*
Pa_52	0.006	0.006	0.000	0.000*	0.000	0.000*
Pa_53	0.000	0.000	0.990	n.a.	n.a.	n.a.
Pa_55	0.000	0.000	0.227	0.000*	0.323	0.000*
Pa_56	0.019	0.018	0.281	0.003*	0.141	0.001*
Pa_59	0.036	0.057	0.000	1.000	0.056	0.342
Pa_60	0.006	0.006	0.694	0.202	0.597	0.005*
Mean	0.027	0.033				

F_ST_, fixation index (Weir, 1996), F_ST_(ENA), fixation index without null alleles (ENA), F_n_, frequency of null alleles, HWE p-value, test for deviation from Hardy-Weinberg equilibrium, *, significant deviation from Hardy-Weinberg equilibrium (P < 0.05), n.a., not analyzed

## Discussion

Several studies describe the development of SSR markers for Norway spruce [8,12]. In the present work we used EST mining of sequences available in databases instead of classical approaches like screening of genomic libraries or fragment enrichment and subsequent cloning. The newly developed markers can be used to complete unsaturated maps, and might be useful in marker assisted breeding and population genetics.

The high number of loci deviating from Hardy-Weinberg equilibrium could partly be explained by the presence of null alleles (Table 3), partly be a result of sampling or selective pressure on the coding regions which is supported by the fact that loci deviating from HWE are not consistent between the two populations. Null alleles can occur due to mutations in primer binding sites and lead to the overestimation of homozygosity as shown by Callen [16]. The presence of such null alleles as estimated in this study may further be confirmed by synthesis of alternative oligonucleotide primer pairs. Increased appearance of homozygotes in some loci supports the hypothesis of selective pressure which might be caused by advantages of recessive or dominant homozygotes known as directional selection. Positive selection was confirmed by outlier detection for two loci which show a high frequency of homozygotes. Both of them are located in coding regions - Pa_42 shows homology to a cysteine protease and Pa_51 is related to a cadmium transporting ATPase. These genes play important roles in various physiological processes, including response to biotic and abiotic stress [17,18]. Therefore, additional data of both populations (e.g. environmental growth) conditions would be of interest. Especially locus Pa_51 may be an interesting target for diversity studies because departure from HWE was only detected in the Mayrhofen population. But although these two loci show considerable degrees of population differentiation, the low F_st _values present in the other loci support previous findings [3,4] that there is no major degree of differentiation among Alpine Norway spruce populations.

The new SSR markers characterized in this study are a good resource for assessing diversity in Alpine spruce populations and might be of further use for applications in forestry. Additionally, 31 monomorphic SSR loci were identified in this study. It is still possible to derive polymorphic data from them when screening Norway spruce populations from different origin, as it has already been shown that flanking regions of monomorphic SSRs show a moderate to high degree of variability [19]. Possible variation within these flanking regions in Norway spruce could serve as alternative source for diversity studies applying different techniques for the identification of variability within these regions.

All variable SSRs are located within ORF sequences and 60% of them gave significant hits to functional genes present in GenBank. A frameshift caused by length variation within an ORF might lead to distortion in the coding region and thus to the abortion of the coded protein. In all of the newly identified EST-SSRs no frameshift is occurring because either, as in 50% of the cases, the SSRs are trinucleotide repeats or the repeat number of polymorphic di- and pentanucleotide repeats alters in such a way that the sum of the basepair variation is a multiple of three.

## Conclusions

We have identified 92 new SSR regions with 48 different repeat motifs in the expressed part of the Norway spruce genome. In two screening populations of 48 individuals each, 23 SSR regions showed a moderate to high degree of polymorphism. Although a high number of SSR markers were already developed for Norway spruce, most of the available SSRs consist of dinucleotide repeats and do not include our tri- and pentanucleotide repeats. We have demonstrated that on the one hand genetic differentiation between distinct stands is low but on the other hand selective forces are likely to have been acting on several genes because deviations from HWE could not only be explained due to null alleles and test for outliers revealed two loci under positive selection. The results gave an insight into the abundant repeat classes and will be of use in analysis of variation linked to expressed genes in Alpine Norway spruce populations as well as evolutionary forces acting at these loci.

## Methods

14,022 Norway Spruce EST sequences were extracted from the NCBI dbEST and screened for SSR sequences with the SciRoKo software program [20]. Only perfect SSRs were searched in two iterations, one for mono- and dinucleotides (at least 4 repeats), and another one for tri-, tetra-, penta- and hexanucleotides (at least 3 repeats). SSR motif containing sequences were subjected to clustering and annotation using the EST2uni pipeline [21]. For clustering, the default settings (30 bp minimum overlap with at least 94% identity for pairwise alignment using BLAST, 93% overlap identity cutoff for assembly using CAP3) were applied. The resulting Unigene Set (contigs and singletons) was compared against three protein databases with BLASTX, in particular NCBI NR, Arabidopsis TAIR7, and Uniprot/Swissprot, using an e-value of 1e-10 as cutoff. The descriptions of the BLAST hits obtained with the different BLAST runs were parsed and merged to yield a descriptive annotation for each unigene. The annotations were attributed with modifiers like "Similar to" or "Highly similar to", depending on the e-value of the alignment with the corresponding BLAST hit.

Plant material used: 16 samples from all over Austria (testing population) and two times 48 individuals from two distinct populations (Gusswerk, Styria [N 47° 44|, E 15° 18|]; Mayrhofen, Tyrol [N 47° 11', E 11° 52']; screening population). DNA was kindly provided by the Federal Research and Training Centre for Forests, Natural Hazards and Landscape (BFW).

60 primer pairs were designed using Primer3 web interface [22] with default settings. PCR reactions were performed in 25 μl volumes containing 50 ng genomic DNA, 1X PCR Buffer (QIAGEN), 1 mM MgCl_2_, 0.2 mM each dNTP, 0.3 μM FAM-labelled forward primer, 0.3 μM reverse primer and 0.625 U HotStar *Taq *polymerase (QIAGEN). The PCR thermal profile consisted of 15 min. initial denaturation at 95 °C followed by 35 cycles denaturation at 95 °C for 50 sec., a primer-specific annealing-temperature (Table 1) for 50 sec., extension at 72 °C for 105 sec., and a final extension at 72 °C for 10 minutes. Fragments amplified in 16 individuals from all over Austria (testing population) and 96 individuals from 2 distinct locations in Austria (screening population) were analyzed on an ABI 3100 Genetic Analyzer. Fragment sizes were extracted using PeakScanner 1.0 (Applied Biosystems, Foster City, California, USA) and allele calls were manually performed in Excel. The testing population was only used to test for polymorphic loci and was not included in further calculations. Number of alleles, effective number of alleles, observed and expected heterozygosities and deviation from Hardy-Weinberg equilibrium were calculated using Genepop 4.0 [23]. Fixation indices and fixation indices excluding null alleles (ENA), as well as estimates of null alleles were calculated with FreeNA [24] and SSR presence in coding regions was analyzed with the GetORF software using default settings. Test for outlier loci were performed with LOSITAN [25] using an infinite allele model with 100.000 simulations, a confidence interval of 0.95 and a subsample size of 75.

## Competing interests

The authors declare that they have no competing interests.

## Authors' contributions

SF participated in the design of the study, carried out the primer design, SSR data analysis and allele calling and drafted the manuscript. AB carried out the PCR and the fragment analysis. AH carried out the population genetic analysis and helped to draft the manuscript. NS participated in the analysis of null alleles and F_ST _values. DK carried out the computational detection of SSRs. All authors read and approved the final manuscript. GGV contributed part of the EST sequences and commented on the manuscript.

## Supplementary Material

Additional file 1 **Monomorphic SSRs_Norway Spruce**. A Microsoft Excel table containing a summary of monomorphic SSRs, accession numbers, primer sequences for amplification, repeat type and repeat number. Click here for file

Additional file 2 **SSR Annotations_Norway spruce**. A Microsoft Excel table showing SSR hits in Genbank, the corresponding accession number and e-value. Click here for file
